# Uncovering the Genome-Wide Transcriptional Responses of the Filamentous Fungus *Aspergillus niger* to Lignocellulose Using RNA Sequencing

**DOI:** 10.1371/journal.pgen.1002875

**Published:** 2012-08-09

**Authors:** Stéphane Delmas, Steven T. Pullan, Sanyasi Gaddipati, Matthew Kokolski, Sunir Malla, Martin J. Blythe, Roger Ibbett, Maria Campbell, Susan Liddell, Aziz Aboobaker, Gregory A. Tucker, David B. Archer

**Affiliations:** 1 School of Biology, University of Nottingham, Nottingham, United Kingdom; 2 School of Biosciences, Sutton Bonington Campus, University of Nottingham, Loughborough, United Kingdom; 3 Deep Seq, Faculty of Medicine and Health Sciences, Queen's Medical Centre, University of Nottingham, Nottingham, United Kingdom; 4 Division of Animal Sciences, Sutton Bonington Campus, University of Nottingham, Loughborough, United Kingdom; Chalmers University of Technology, Sweden

## Abstract

A key challenge in the production of second generation biofuels is the conversion of lignocellulosic substrates into fermentable sugars. Enzymes, particularly those from fungi, are a central part of this process, and many have been isolated and characterised. However, relatively little is known of how fungi respond to lignocellulose and produce the enzymes necessary for dis-assembly of plant biomass. We studied the physiological response of the fungus *Aspergillus niger* when exposed to wheat straw as a model lignocellulosic substrate. Using RNA sequencing we showed that, 24 hours after exposure to straw, gene expression of known and presumptive plant cell wall–degrading enzymes represents a huge investment for the cells (about 20% of the total mRNA). Our results also uncovered new esterases and surface interacting proteins that might form part of the fungal arsenal of enzymes for the degradation of plant biomass. Using transcription factor deletion mutants (*xlnR* and *creA*) to study the response to both lignocellulosic substrates and low carbon source concentrations, we showed that a subset of genes coding for degradative enzymes is induced by starvation. Our data support a model whereby this subset of enzymes plays a scouting role under starvation conditions, testing for available complex polysaccharides and liberating inducing sugars, that triggers the subsequent induction of the majority of hydrolases. We also showed that antisense transcripts are abundant and that their expression can be regulated by growth conditions.

## Author Summary

The aim of second generation biofuels is to produce fuels from non-food crops and agricultural wastes such as straw. A key, and often limiting, step is the extraction of simple sugars (saccharification) from the complex plant materials. This typically requires the use of fungal enzymes. Many such enzymes have been isolated and characterised, but less is known about how fungi naturally utilise their array of enzymes and what other strategies they employ in degrading plant material. In this study, we show that the filamentous fungus *Aspergillus* niger deploys a large number of plant cell wall-degrading enzymes when using wheat straw as its carbon source. Our results identify several other types of proteins that may play a role in this process, and thereby offer applications in the improvement of current saccharification processes. In addition, we show that wheat straw itself is not initially detected by the fungus and, instead, the onset of carbon starvation triggers the release of a small subset of degradative enzymes. These enzymes might play a scouting role, to sense the presence of plant cell walls and initiate degradation on a small scale, in turn releasing sugars that cause the fungus to express its full degradative arsenal.

## Introduction

The conversion of cellulose and hemicellulose, from non-food crop sources into fermentable sugars is one of the key challenges in the production of second generation biofuels. Fungi are the predominant source of enzymes currently being used on an industrial scale for this purpose [1], [2]. Many relevant enzymes have been isolated and characterised for functionality [3], [4]. The overall aim of our study was to look beyond the simple array of hydrolytic enzymes produced by fungi and to understand the strategies that fungi employ to degrade complex polysaccharides. This approach may provide novel insights into the development of strategies for the production of second generation biofuels.

*Aspergillus niger* is a filamentous, black-spored fungus that has been used in many industrial processes, including the production of enzymes, food products and pharmaceuticals [5]. This historical importance has led to the development of a wide array of genetic tools [6]. These include a variety of mutagenesis systems, both targeted [7] and random [8], highly tuneable expression systems [9], and complete genome sequences for both the enzyme-producing industrial strain CBS 513.88 [10] and the citric acid-producing strain, ATCC 1015 [11]. The Carbohydrate-Active Enzymes Database (http://www.cazy.org/ [12]) identifies the CBS 513.88 genome as encoding 281 putative polysaccharide degrading enzymes, that represent 61 different enzyme families. Thus, the genome of *A. niger* encodes one of the most diverse CAZy enzyme arsenals among currently sequenced fungal genomes. The availability of DNA microarrays for *A. niger* has led to discoveries in the areas of protein secretion [13] and of global transcriptional responses to simple sugars such as glucose, xylose and glycerol [14]–[16]. This genomic information is complemented by studies that have elucidated some of the basic molecular pathways by which hydrolytic enzyme production and sugar metabolism are regulated in *A. niger* [17], [18]. Furthermore, genome-wide approaches provide a basis for comparability with other fungal species [19], [20]. This wealth of background knowledge of *A. niger* and closely-related species, and their responses to monomeric sugars and simple polysaccharides, provides an excellent foundation on which to build more complete studies of growth on more complex, industrially relevant substrates. Measuring gene expression of *Neurospora crassa* during growth on *Miscanthus* [21], and transcriptional changes when *A. niger* is exposed to sugar cane bagasse [13] using microarray technology have previously provided important insights into degradation of those substrates.

Wheat straw is one of the most attractive potential feed stocks for biofuel production. It is a co-product of cereal grain production and is available in significant quantities; for example, in the order of 10 million tonnes are produced in the UK each year [22]. This study aims to gain a thorough understanding of the mechanisms employed by *A. niger* to degrade and grow upon this complex lignocellulosic substrate, beginning with the transcriptional changes associated with exposure to wheat straw compared to simple sugars. Our data were acquired using Next Generation RNA-sequencing (RNA-seq) technology which provides a wealth of information for the confirmation of gene predictions from both published genomes, as well as in identifying alternative splicing patterns, novel genes/exons, transcription start/end points and antisense (AS) transcripts.

## Results/Discussion

### Saccharification of wheat straw by *A. niger*

**Figure 1:**
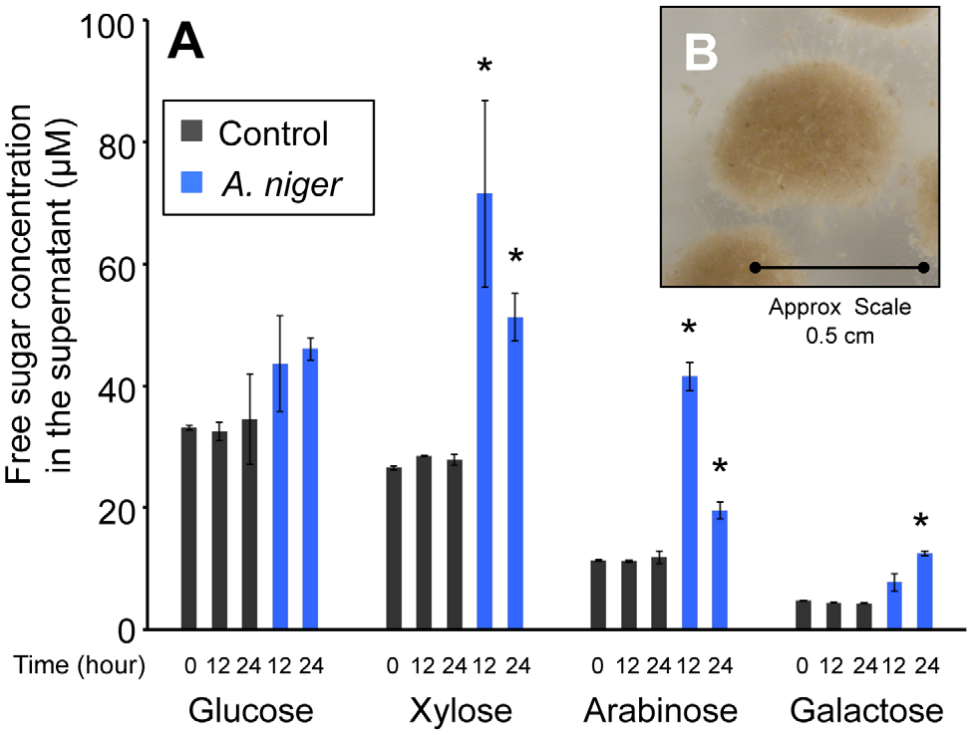
Free sugars in the straw media. (A) The monomeric sugar content of culture supernatants was analysed by HPLC at 0, 12 and 24 h after the transfer of the mycelia to the straw media. Each bar represents the mean +/− the standard deviation of values from three independent experiments where black represents control cultures and blue represents cultures containing *A. niger*. The asterisk indicates p-values<0.05 relative to the control culture at the corresponding time by unpaired t-test. (B) *A. niger* mycelial clump from a culture grown for 24 hours in minimum media containing straw as sole carbon source.

The wheat straw used was composed of 37±1.69% cellulose and 32±1.2% hemicelluloses, 22±0.1% lignin and, after ball milling, the substrate retained 25±0.76% crystallinity (data are the mean of three replicates and are shown ± standard deviation). In order to determine the time at which degradation of the wheat straw by *A. niger* had begun to take place, the monomeric sugar content of the culture supernatant was analysed by HPLC. Figure 1A shows that, prior to inoculation, in minimal media containing 1% straw, the concentration of free monomeric sugar present in the liquid fraction was 76±0.9 µM. After 12 h of incubation, control samples, which had not been inoculated with *A. niger*, contained similar levels of each sugar. In the *A. niger* cultures, the total concentration of free monomeric sugar present in the liquid fraction increased to 166±26.9 µM, showing that degradation of wheat straw polysaccharides had begun to take place. There were also changes detected in the proportions of individual sugars (Figure 1A). Xylose, arabinose and galactose levels were increased by a statistically significant level, whilst glucose levels were not. Two non-exclusive hypotheses could explain this observation; i) the hemicellulose fraction of the lignocelluloses substrate is degraded first, and/or ii) glucose is preferentially imported by the fungus. Indeed a requirement for glucose depletion prior to xylose utilisation, when both sugars are present, has been observed in *Aspergillus nidulans* [23]. After 24 h of incubation, levels of free sugar had not increased any further suggesting that the balance of degradation and sugar uptake had reached a steady state. RT-PCR showed that transcription of several genes encoding glycoside hydrolases, (endoglucanase; *eglA*, cellobiohydrolase; *cbhA* and the endoxylanases; *xynA* and *xynB*) that are transcriptionally activated in response to xylose by the xylanolytic regulator XlnR, were highly induced at this point, when compared to expression in the Glucose 48 h cultures (data not shown). These two time-points were therefore chosen for RNA-seq analysis (Glucose 48 h and Straw 24 h). After 24 h incubation in the straw media, the particles of straw were in intimate association with *A.niger* mycelia (Figure 1B). It is possible therefore that the responses seen are due not only to the presence of inducing molecules, but also due to the physical interaction between the fungal mycelia and the straw.

To investigate the repressive effect of glucose on expression of degradative enzymes, glucose was added exogenously to the wheat straw-incubated 24 h cultures to a final concentration of 1% (w/v). Samples were taken for RT-PCR analysis after 30 min, 1, 2 and 5 h. For all genes tested the levels of hydrolase expression decreased over this time course, reaching for most of the genes a similar level to that seen in the Glucose 48 h cultures after 5 hours of exposure to the exogenously added glucose (data not shown). Therefore, 5 hours after the addition of glucose to the straw cultures was selected as the final time point for RNA-seq analysis (Straw+Glucose 5 h). These represent three physiologically distinct conditions; long term growth under glucose repressing conditions, growth in the presence of an inducing lignocellulosic substrate and growth in the presence of the inducing substrate and glucose simultaneously. RNA was extracted from triplicate independent cultures in each condition for RNA-seq analysis.

### The wheat straw-induced transcriptome of *A. niger*

Reads were mapped to the *A. niger* ATCC 1015 genome sequence [11] as it is phylogenetically very close (based on the β-tubulin sequence) to the N402 strain used in this study, and RPKMs (Reads Per Kilobase of exon model per Million mapped reads) were calculated for each annotated gene. The ATCC 1015 gene model is thought to under-predict the true number of genes present in the *A. niger* genome [11] so, in order to extract the maximum amount of data from our transcriptome sequencing, reads were also mapped to the CBS 513.88 sequence [10], which has a greater number of predicted genes. Approximately 2.5% more reads were successfully mapped to the ATCC 1015 genome than the CBS 513.88, reflecting the closer relationship between this strain and N402 used in this study. The CBS 513.88 genome model contains 4213 genes not included within the ATCC 1015 genome model and 939 of these genes were found to have an RPKM of 1 or more in at least one of the conditions tested in our transcriptome and are therefore very likely to be present within the ATCC 1015 genome also. A full list of the gene expression for the 4213 genes is included in supplementary material (Table S1). Eight of these genes encoded potential polysaccharide degrading enzymes or demonstrated a transcriptional pattern in the CBS 513.8 of interest to this study and so were added to the ATCC 1015 gene model and their RPKM values from the ATCC 1015 mapping were calculated.

RPKM values for all genes were calculated for each of the separate biological replicates, as well as for the combined mapping of all three, (Table S2). Table S3 shows that inter-replicate reproducibility was extremely high (R-squared values of >0.9). The results shown within the main text are from the combined mapping scores. Three statistical significance tests were applied to changes in gene expression, the Likelihood Ratio Test [24], Fisher's Exact Test [25], and an MA-plot-based method with Random Sampling model [26]. The results of these tests for each gene are listed in Table S2. All gene inductions discussed within the text had a p-value of <0.001 for all three statistical tests.

### Expression of CAZy genes

Deconstruction of plant cell wall polysaccharides is mediated by enzymes of three major classes: Glycoside Hydrolases (GH), Carbohydrate Esterases (CE) and Polysaccharide Lyases (PL). The Carbohydrate Active Enzyme database (CAZy - http://www.cazy.org) subdivides enzymes within these three classes into families based on their related activity and sequence. CAZy defines 281 of these enzymes within the CBS 513.88 genome, all but two of which are also predicted in the ATCC 1015 genome model (CBS 513.88 annotation: An14g07390 and An14g07420). The ATCC 1015 genome encodes 246 predicted GHs, representing 51 families; 25 CEs representing 9 families and 8 PLs representing 2 families.

**Figure 2:**
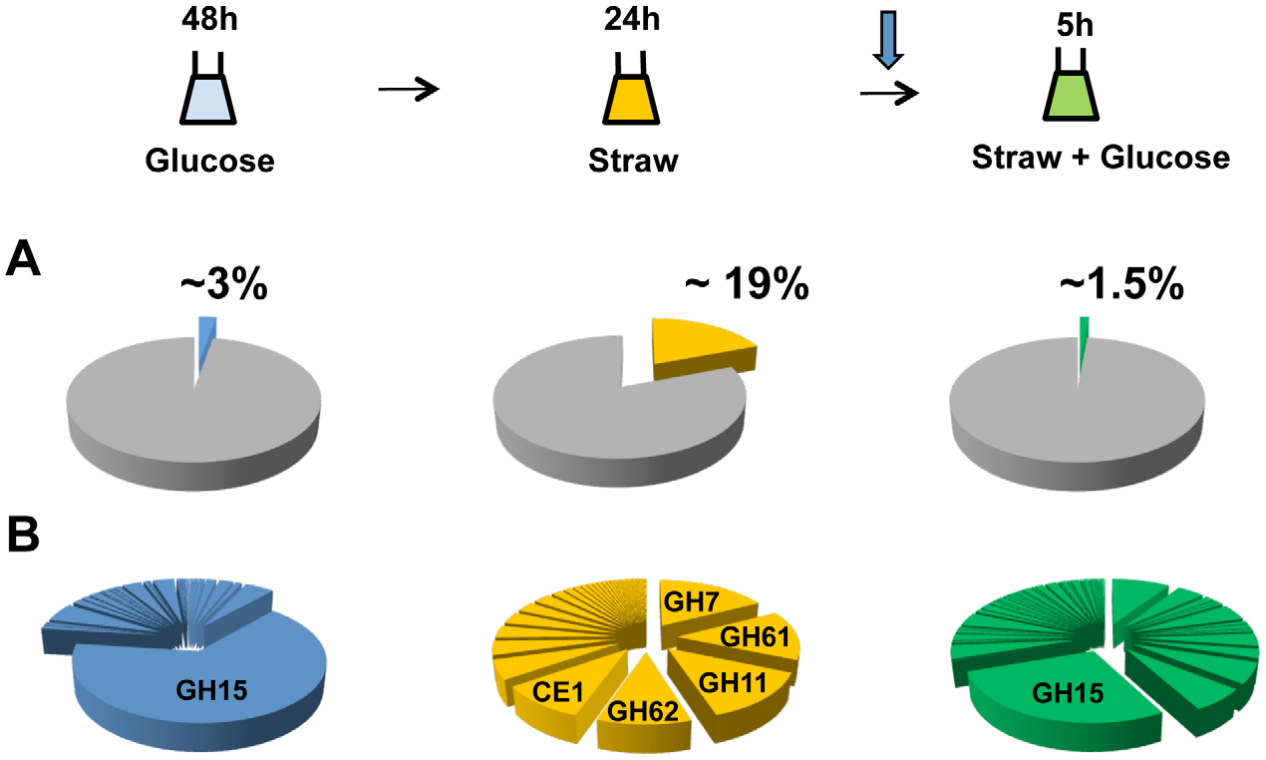
CAZy gene expression. The sampling conditions are shown for the RNA-seq study. Cells were grown in glucose (48 h), washed and transferred to straw (24 h) and then glucose was added (downward arrow) followed by a further 5 h incubation. (A) Percentage of total mRNA (calculated from RPKM values) represented by CAZy family genes from each condition of the transcriptome study. (B) Proportions of total of CAZY gene mRNA from each enzyme family. The families are listed in decreasing order of expression in the Straw 24 h condition.

After 48 hours of growth in minimal media with 1% glucose, CAZy genes represent approximately 3 percent of total mRNA, with the glucoamylase *glaA* (GH15 family) accounting for the majority (over 65 percent) of this (Figure 2A). SDS-PAGE of the culture supernatant revealed the presence of a highly predominant protein band, which was identified by tandem MS as GlaA (data not shown). Twenty-four hours after the mycelia were transferred to straw, expression of CAZy genes made up more than 19 percent of total mRNA. This is a strong over-representation of the CAZy group of genes, as they represent only ∼2.5 percent of the coding genome. Thirty of the induced CAZy genes reached an expression level above 50 RPKM. They represent 14 families of GH, 2 of CE and 1 of PL (Table 1).

**Table 1:** Straw-induced CAZy genes.

Gene ID		CAZy	CADRE Annotation	RPKM		
ATCC1015	CBS 513.88	Family		Glucose 48 h	Straw 24 h	Straw+Glucose 5 h
53159	An07g09330	GH 7	1,4-beta-D-glucan cellobiohydrolase - *cbhA*	6.2	4137.0	21.5
211595	An12g04610	GH 61	Partial similarity to endoglucanase IV *egl4* -*Trichoderma reesei*	3.3	3389.9	54.3
52071	An01g00780	GH 11	Endo-1,4-xylanase - *xynB*	4.4	3015.4	14.9
55136	An03g00960	GH 62	1,4-beta-D-arabinoxylan arabinofuranohydrolase - *axhA*	4.1	2521.9	9.3
211544	An12g05010	CE 1	Acetyl xylan esterase - *axeA*	1.6	1514.1	5.6
179265	An11g00200	GH 3	Probable beta-glucosidase - *bglM*	1.1	924.6	16.7
43785	An12g02550	CE 1	Probable feruloyl esterase - *faeC*	1.0	902.9	2.5
56619	An14g05800	GH 67	Alpha-glucuronidase - *aguA*	1.4	828.3	6.7
57436	An03g00940	GH 10	Endo-1,4-beta-xylanase - *xynA*	0.9	713.0	4.6
54490	An12g02220	GH 6	Probable 1,4-beta-cellobiosidase - *cbhC*	0.7	611.5	5.7
211053	An14g02760	GH 12	Endoglucanase - *eglA*	1.1	510.8	10.8
133986	An08g01760	GH 6	Similarity to cellobiohydrolase - *Acremonium cellulolyticus*	0.4	346.5	4.9
52011	An01g03340	GH 12	Probable xyloglucan-specific endo-beta-1,4-glucanase - *xgeA*	1.3	334.4	4.0
183088	An15g04550	GH 11	Endo-1,4-beta-xylanase	0.6	327.4	5.0
209376	An07g08950	GH 5	Endoglucanase - *eglC*	0.3	316.2	2.1
125000	An01g04560	GH 16	Similarity to mixed-linked glucanase MLG1 - *Cochliobolus carbonum*	2.0	302.0	58.6
56782	An18g03570	GH 3	Probable beta-glucosidase - *bglA*	2.6	275.8	31.5
47677	An08g01900	GH 43	Similarity to 1,4-beta-xylosidase - *Butyrivibrio fibrisolvens*	0.4	209.7	1.9
205580	An01g11670	GH 5	Similarity to endo-beta-1,4-glucanase - *Aspergillus nidulans*	0.2	208.2	8.4
45801	An03g00500	GH 30	Similarity to diglycosidase related protein -*Aspergillus fumigatus*	5.1	147.8	24.5
51773	An01g11660	GH 7	1,4-beta-D-glucan cellobiohydrolase - *cbhB*	0.3	121.1	102.7
135787	An07g08940	CE 16	Similarity to acetyl-esterase I - *Aspergillus aculeatus*	0.1	118.7	0.5
55419	An01g04880	GH 31	alpha-glucosidase II -*axlB*	1.8	109.7	0.7
202490	An18g04100	GH 5	Probable glucan 1,3-beta-glucosidase - *exgA*	0.4	99.0	15.2
210947	An14g01130	PL 4	Probable rhamnogalacturonate lyase - *rglA*	0.2	86.2	1.7
43342	An09g03300	GH 31	alpha-xylosidase - *axlA*	3.7	78.8	14.1
203143	An09g01190	GH 43	Endo 1,5-alpha-arabinanase - *abnA*	0.1	74.0	2.2
197735	An02g10550	GH 43	Probable endo-1,5-alpha-arabinanase - *abnC*	0.4	61.7	11.1
50997	An17g00300	GH 3	Similarity to xylosidase-arabinosidase *xarB* - *Thermoanaerobacter ethanolicus*	0.4	58.3	1.2
This Study	An02g02540	CE 16	Similarity to acetyl-esterase I - *Aspergillus aculeatus*	0.9	56.4	0.5

CAZy genes strongly induced (≥20 x) and expressed (≥50 RPKM) after 24 h in Straw. An02g02540 has not been annotated in the ATCC 1015 genome but was found on chromosome 4_2 (5′-669026 - 667767-3′). Annotation are from CADRE [66] (http://www.cadre-genomes.org.uk/index.html). RPKM values are from the combined mapping of three biologically-independent samples under each condition, values for each individual sample are listed in Table S2. All genes listed showed a statistically significant induction when switched from glucose to straw (p>0.001).

The diverse categories of CAZy genes expressed during exposure to straw reflect the complexity of the carbohydrates present within the substrate. However, it is interesting to note that around 65 percent of the mRNA from the CAZy group at this time-point is from genes encoding just 5 families of enzyme, GH7, 11, 61 and 62 (cellobiohydrolases, xylanases, polysaccharide monooxygenases and arabinofuranosidases, respectively) and CE1 (acetyl xylan esterases) (Figure 2B and Table 1). Proteins from each of these categories, except GH61, were also identified within culture supernatants by SDS-PAGE and tandem MS (data not shown). The fact we did not identify any GH61 proteins, that play a role in the oxidative cleavage of recalcitrant plant biomass [27]–[29], amongst the major bands could be due to a discrepancy between transcript and protein levels, or simply a technical issue in detection, such as the protein not staining well, or the protein being attached to the substrate or the fungal membrane, whilst only the supernatant was analysed. Based on transcript abundance, the 5 categories of encoded enzyme might provide the bulk of activities required for the degradation of straw. Catabolite repression, by addition of glucose to the straw cultures, exerts strong repression upon CAZy gene expression; after 5 hours, CAZy gene mRNA is reduced to only 1% of total mRNA. The glucoamylase *glaA* (GH15) becoming again the most expressed CAZy gene under this condition (Figure 2B).

### Expression of non-CAZy genes

Twenty-eight genes that were transcriptionally induced more than 20-fold, and reached an RPKM of greater than 50 after the switch to straw, do not encode hydrolases as classified by CAZy. These genes are listed in Table 2 and 23 of them fall into four broad functional categories: lipases/esterases, surface interacting proteins, enzymes of carbon and nitrogen metabolism and transporters.

**Table 2:** Straw-induced non-CAZy genes.

Gene ID		CADRE Annotation	RPKM		
AT15CC10	CBS 513.88		Glucose 48 h	Straw 24 h	Straw+Glucose 5 h
		**Esterases & Lipases**			
173684	An02g09690	Similarity to lipase I precursor TFLI - *Geotrichum fermentans*	1.0	1728.9	6.6
51662	An09g00120	Ferulic acid esterase A FaeA	0.9	1100.5	3.7
50877	An13g01880	Esterase EstA	2.9	317.6	3.0
210730	An16g01880	Similarity to lysophospholipase - *Aspergillus foetidus*	0.6	203.7	2.5
54865	Not Present	GDSL lipase/acylhydrolase family protein - *Aspergillus fumigatus*	0.3	168.0	0.7
This Study	An03g06560	Similarity to triacylglycerol lipase Lip2 - *Candida rugosa*	0.2	84.4	0.8
53620	An16g03700	Similarity to phospholipase B - *Aspergillus oryzae*	1.7	62.5	62.5
		**Surface-interacting Proteins**			
128530	An07g03340	Hydrophobin Hyp1	3.5	380.4	423.2
This Study	An08g09880	Hydrophobin HbD	0.0	138.5	29.3
188224	An09g00840	Similarity to HsbA - *Aspergillus oryzae*	0.3	118.6	1.0
54125	An18g02730	Similarity to transmembrane protein PTH11 - *Magnaporthe grisea*	1.9	70.5	4.1
		**Enzymes of Carbon and Nitrogen Metabolism**			
51997	An01g03740	D-xylose reductase XyrA	1.1	448.7	2.4
52460	An02g13750	Similarity to glutaminase A *GtaA - Aspergillus oryzae*	2.2	213.7	14.7
40496	An15g02410	Similarity to nitrogen metabolic repression regulator hNmrr - *Homo sapiens*	0.3	132.6	45.3
40740	An15g05990	Similarity to D-arabinitol 2-dehydrogenase ARDH - *Pichia stipitis*	0.6	69.6	22.6
56084	An11g10890	Similarity to UDP-glucose 4-epimerase Gal10 - *Saccharomyces cerevisiae*	2.1	51.2	0.9
		**Transporters**			
56643	An12g09270	Lactose permease - Lac12	3.3	273.4	34.1
38375	An08g04040	Similarity to quinate transport protein QutD - *Aspergillus nidulans*	1.7	141.9	2.9
55668	An06g00560	Similarity to hexose transporter Hxt13 - *Saccharomyces cerevisiae*	0.6	124.0	3.7
180069	An07g02540	Similarity to carboxylic acid transport protein Jen1 - *Saccharomyces cerevisiae*	1.5	108.6	2.0
197549	An02g08230	Similarity to high affinity glucose transporter HGT1 - *Kluyveromyces lactis*	1.0	105.4	0.8
54095	An18g01700	Similarity to quinate transport protein *GutD* - *Aspergillus nidulans*	0.2	98.6	1.8
54838	An13g03110	Similarity to high-affinity nicotinic acid permease Tna1 - *Saccharomyces cerevisiae*	0.6	66.6	0.7
		**Others**			
120161	An18g05500	Similarity to mitochondrial ceramidase AAF86240.1 - *Homo sapiens*	1.6	144.9	1.1
42809	An18g03380	Similarity to mitochondrial thioredoxin Trx3 - *Saccharomyces cerevisiae*	2.1	98.0	2.8
180489	An07g00070	Similarity to hypothetical protein EAL85123.1 - *Aspergillus fumigatus*	1.1	66.1	25.5
53013	An11g07040	Similarity to EST an_2779 - *Aspergillus niger*	0.3	65.5	1.1
43786	An12g02560	Similarity to protein-tyrosine phosphatase SH-PTP2 - *Rattus norvegicus*	1.8	51.3	36.1

Non-CAZy genes strongly induced (≥20 x) and expressed (≥50 RPKM) after 24 h in Straw. An03g06560 and An08g09880 have not been annotated in the ATCC 1015 genome but were found on chromosome 6_1 (5′-1494949 - 1403261-3′) and 8_2 (5′-2365138 - 2364987 - 3′) respectively. Annotation are from CADRE [66] (http://www.cadre-genomes.org.uk/index.html). RPKM values are from the combined mapping of three biologically-independent samples under each condition, values for each individual sample are listed in Table S2. All genes listed showed a statistically significant induction when switched from glucose to straw (p>0.001).

#### Lipases and esterases

Seven putative lipases or esterases not classified as CEs by CAZy were strongly induced after the transfer to straw, and 6 of these were repressed by addition of glucose (Table 2). The esterase EstA (TID_50877) was shown to be regulated by XlnR [30], and we identified it by SDS-PAGE and tandem MS as being one of the major proteins present in the supernatant after 24 hours in straw, while the ferulic acid esterase (FaeA) has been shown to have activity against wheat arabinoxylan [31]. Whilst the others have not previously been associated with polysaccharide degradation, their co-expression alongside the well characterised *estA* and *faeA*, raises the possibility that these enzymes may be involved in the saccharification of wheat straw lignocellulose.

#### Surface-interacting proteins

Two genes encoding hydrophobin family proteins and one hydrophobic surface binding protein (HsbA) were strongly induced by the switch from glucose to straw (Table 2). The gene *hyp1* is not repressed by the re-addition of glucose to the culture, but *hfbD* and and *hsbA* were strongly repressed; their transcriptional profile is therefore similar to many of the genes of the CAZy group. Hydrophobins are amphipathic, surface-active proteins produced by filamentous fungi, with numerous biological functions, often relating to mycelial interactions with solid surfaces [32]. RolA (homologous to Hyp1) and HsbA of *Aspergillus oryzae* have been shown to associate with the synthetic polyester polybutylene succinate-coadipate and promote its degradation through the recruitment of a specific polyesterase [33], [34]. It is striking therefore that *A. niger* genes that encode proteins bearing homology to each, are highly induced by the presence of straw, suggesting that these proteins could have a role in recruiting degradative enzymes to the straw surface. The last gene in this group TID_54125 encodes a G-protein couple receptor homologous to Pth11p from the rice pathogen *Magnaporthe grisea*. Pth11p is required for signalling in appressorium formation [35] through the sensing of the plant host surface via both hydrophobicity and the presence of cutin monomers (which are also a component of the wheat straw cuticula [36]). The induction of the *A. niger* homologue of *pth11* in the presence of straw suggests that a similar strategy could perhaps be employed by *A. niger* in the sensing of solid substrate surfaces.

#### Carbon metabolism

The increased expression of the xylose reductase *xyrA* and other genes of the xylose utilisation pathway, such as xylitol dehydrogenase (TID_203198) and D-xylulokinase (TID_209771) (both induced approximately 10-fold, Table S2), along with decreased expression of glycolytic pathway enzymes (e.g. phosphofructokinase, TID_54093, repressed 5-fold), suggests that 24 hours after the transfer to straw, pentose sugars are the prominent carbon source available. These results, and the fact that *A. niger* is an efficient xylan-degrading organism [37], support the first of the hypotheses discussed earlier, that the reason xylose is the predominant free sugar observed after 24 hours incubation of wheat straw with *A. niger* (Figure 1A) is that the hemicellulose fraction of the straw is the first to be degraded, rather than being due to a greater rate of glucose uptake by the fungus.

### The degradative response is sequential and triggered by carbon starvation

To establish the order of induction of the genes that were highly expressed by 24 h, a time-course experiment was performed. RNA was extracted 0.5, 1, 2, 3, 6, 9 and 12 h after the switch from glucose to straw, and the expression of genes of interest was measured using RT-PCR (Figure S1). The results show the cellobiohydrolase *cbhB* to be induced after 6 hours of exposure to straw, whilst *cbhA* and *GH61a* do not appear to be induced until the 9 hour time point. These timings were verified, and shown statistically significant, using quantitative RT-PCR (Figure S2). To investigate the regulatory basis for the differential response of these genes, expression under the glucose and straw conditions was examined in strains deleted for either the gene encoding the xylanolytic activator XlnR, that mediates xylose-induction of some GHs and esterases, or CreA, which mediates wide-domain carbon catabolite repression [38]. All three of the glycosyl hydrolases showed a statistically significant (p-value of >0.01 in an equal variance, one-tailed T-test) dependence upon XlnR for maximal induction at 24 h in straw (Figure S3), which is typical of hydrolases in *A. niger* [39], [40]. Interestingly, a smaller scale induction of *cbhB* in the straw media was still observed in the *ΔxlnR* strain. This induction is mediated by alleviation of CreA repression, since in the *ΔcreA* strain, *cbhB* was expressed at a significantly higher level than the wild-type during growth on glucose, as assessed by qRT-PCR, whilst *cbhA* and *GH61a* were not (Figure S4). This observation led to the hypothesis that the earlier induction seen for *cbhB* is triggered, not by the presence of an inducing sugar such as xylose, but instead by the absence of an available carbon source. Transferring mycelia from glucose (48 h) to media completely devoid of carbon source for 24 h had a significant inductive effect upon *cbhB* expression, whereas it did not induce *cbhA* or *GH61a* (Figure S5). As only a very low concentration of free xylose is present in the straw media until several hours after incubation with *A. niger* (Figure 1A), this might explain why the induction of *cbhB* occurs earlier than the induction of *cbhA* or *GH61a*.

CbhA and CbhB are the only two GH7-family cellobiohydrolases in *A. niger*. CbhB contains a family 1 Carbohydrate-Binding-Module (CBM), whilst CbhA has no CBM [41]. CBMs aid the attachment of the enzymes containing them to the complex polysaccharide surfaces of intact cell walls [42], [43]. It could be speculated that the CBM-containing enzyme is induced early because it plays an important role in targeting the relatively intact plant cell wall. Whilst the enzyme lacking the CBM is induced later, once soluble oligosaccharides have been released. Six other genes that were induced after 24 h in straw also encoded proteins containing CBM domains. Two of these genes, TID_205580 (encoding a member of the GH family 5, containing a CBM 1) and *abfB* (an arabinofuranosidase containing a CBM 42), showed the same pattern of expression and regulation as *cbhB*; i.e. de-repression in a *ΔcreA* strain, induction 6 h after transfer to straw, and induction by carbon source starvation (Figures S3, S5 and S6). This CreA de-repression-dependent/XlnR activation-independent mechanism of induction may allow a subset of hydrolases that are targeted more specifically to the intact plant cell wall to play a “scouting” role under carbon starvation conditions, testing for available complex polysaccharides and liberating small quantities of sugar and oligosaccharides which trigger the subsequent larger scale induction of the genes themselves and the remaining majority of hydrolases by XlnR. Such a system would allow the organism to probe the surrounding environment for complex substrates when undergoing carbon starvation, without over-committing resources, until the presence of a degradable substrate is sensed from the release of inducing sugars. The expression of the “scouting” enzymes themselves, as well as the majority of hydrolases, which are completely dependent upon an active form of XlnR for expression, are then induced to extremely high levels (around one-fifth of the total mRNA inside the cell 24 hour after the transfer to straw, Figure 2A). None of the genes identified here as being induced early are predicted to encode enzymatic activities capable of releasing xylose from wheat straw, which would lead to XlnR activation. However, our preliminary data (not shown) indicates that the genes shown here are only part of a larger subset, which may encode such activity. Defining the full subset of hydrolase genes that are induced concurrently with *cbhB* is the subject of ongoing study.

Of the non-CAZy genes that are responsive to straw, both the putative lipase gene (TID_173684) and esterase, *estA* (TID_51662), are induced by the 6 h time point (Figure S1); they therefore form part of the early response, along with *cbhB*, *abfB* and the GH5 family member TID_205580. The lipase-encoding gene shares the same CreA-dependent expression pattern as *cbhB* (Figure S7B). The genes encoding the Pth11 homologue and HsbA are also induced by a lack of carbon source but their induction shows dependence upon neither XlnR nor CreA (data not shown). The extremely early expression of the homologue of *pth11*, after 3 h, may indicate a role in the early stages of the signalling pathway and is the subject of current investigation. The concurrent timing of expression of *hsbA* with the majority of hydrolases, at 9 h, raises the possibility of a role in recruiting hydrolases to the surface of the straw, i.e. analogous to the recruitment of degradative enzymes to the surface of certain plastics [33], [34]. The gene encoding the hydrophobin HfbD was induced later, at 12 h. The possible functionality of surface binding proteins in the recognition of surfaces, and the possibility that those proteins recruit hydrolytic enzymes to the surface, is intriguing and may provide new avenues to enhance the action of hydrolases in the saccharification of biomass in the generation of biofuels and the synthesis of other chemicals within a bio-based economy.

**Figure 3:**
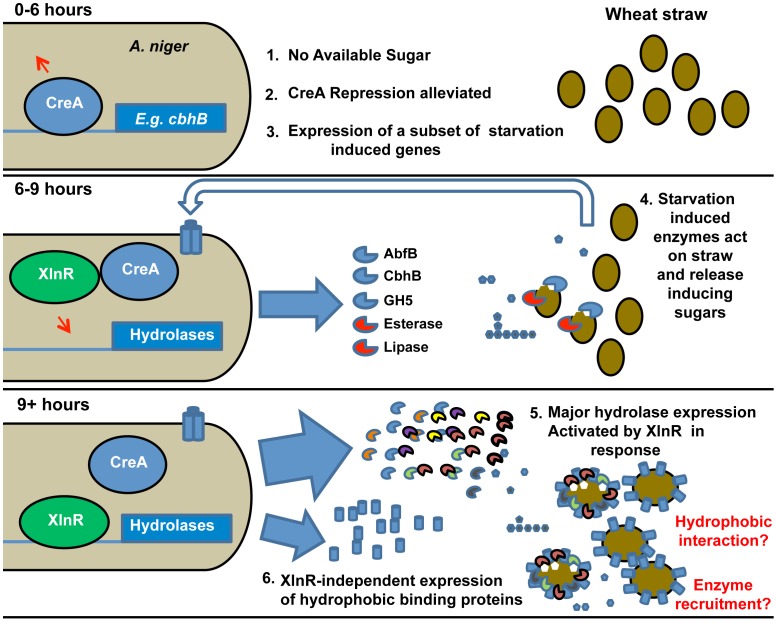
Induction model based on the sequential expression of responsive genes. The sequence of events is illustrated and key events are numbered. The upper panel represents the transcriptional events in *A. niger* upon exposure (0–6 h) to straw represented by filled ovals. Lack of easily-available carbon source leads to the alleviation of CreA repression (represented by the arrow above CreA) and induction of a subset of starvation-induced genes represented by *cbhB*. At 6–9 h exposure to straw (middle panel), the expressed hydrolases and other enzymes (examples named in the Figure.) act upon the wheat straw, releasing small quantities of inducing sugars such as xylose (filled pentamer) as well as glucose (filled hexamer). Transporters for the sugars are induced (indicated by the trans-membrane cylinders and un-filled large arrow). By 9 hours (lower panel) the presence of xylose has caused activation of XlnR and, thereby, large scale expression of hydrolases genes. Also induced by 9 hours, in an XlnR-independent manner, is the hydrophobic binding protein HsbA. The hydrophobin HfbD is induced by 12 hours. A physical association of hydrophobic binding proteins with straw and degradative enzymes is hypothesised and represented. Note that the functionality of XlnR and CreA is indicated by attachment to recognition sequences in target promoters and is meant only to indicate the functional control of those promoters by the transcription factors. Modifications to those transcription factors (e.g. phosphorylation of XlnR in *A. oryzae* [65]) may occur without necessarily implying that their location changes.

The mechanism by which the presence of a complex substrate, which cannot be imported into the cell, is signalled and triggers expression of the requisite hydrolytic enzyme mix, is widely debated and may vary with fungal species. The predominant induction model in fungal systems [21], [44] proposes that general basal expression of small quantities of hydrolase begins the degradation of complex polysaccharides, thereby producing inducing compounds that elicit the full transcriptional response. It has alternatively been suggested that some relevant genes are induced by carbon source depletion, and that the derived enzymes might play a foraging role, under starvation conditions [45]. Temporally differential expression of complex polysaccharide degrading enzymes and the presence of “scouting” enzymes that do not require the presence of a substrate-derived inducer for expression, but work to release inducing molecules and trigger a larger degradative response, has also been observed for the *A. niger* response to pectin [46], [47] and starch [48] perhaps indicating a conserved response pattern to insoluble substrates. Our data support this model for lignocellulose and we take it further by proposing a succession of events that not only includes the timing of expression of genes encoding hydrolases but also involves sensing proteins and the possibility that surface-binding proteins serve as a scaffold for recruitment of hydrolases. In summary the overall strategy appears to be an induction of a specific, small scale, sensory response by carbon source starvation, mediated at least partially by alleviation of CreA-dependent catabolite repression. This leads, in the event of successful liberation of free sugars from the wheat straw substrate, to the full scale degradative response, which is activated by XlnR (Figure 3).

### Antisense transcription

Natural antisense transcripts (NATs) are RNAs transcribed from a region of the genome that lies antisense (AS) to a gene. They have been found in a number of organisms, including several fungi [49]–[51], and play various regulatory roles [52]. The number of reads that fell upon the AS strand was counted for each gene in our study and AS RPKM values were calculated in each condition. AS reads accounted for approximately 2% of total reads under the conditions tested (2.41, 1.94 and 2.04% in Glucose 48 h, Straw 24 h and Straw+Glucose 5 h, respectively). Although the vast majority of genes had just a few associated AS transcripts, 521 genes had an AS RPKM of greater than 1 and up to about 120 (Figure S6 and Table S4).

**Figure 4:**
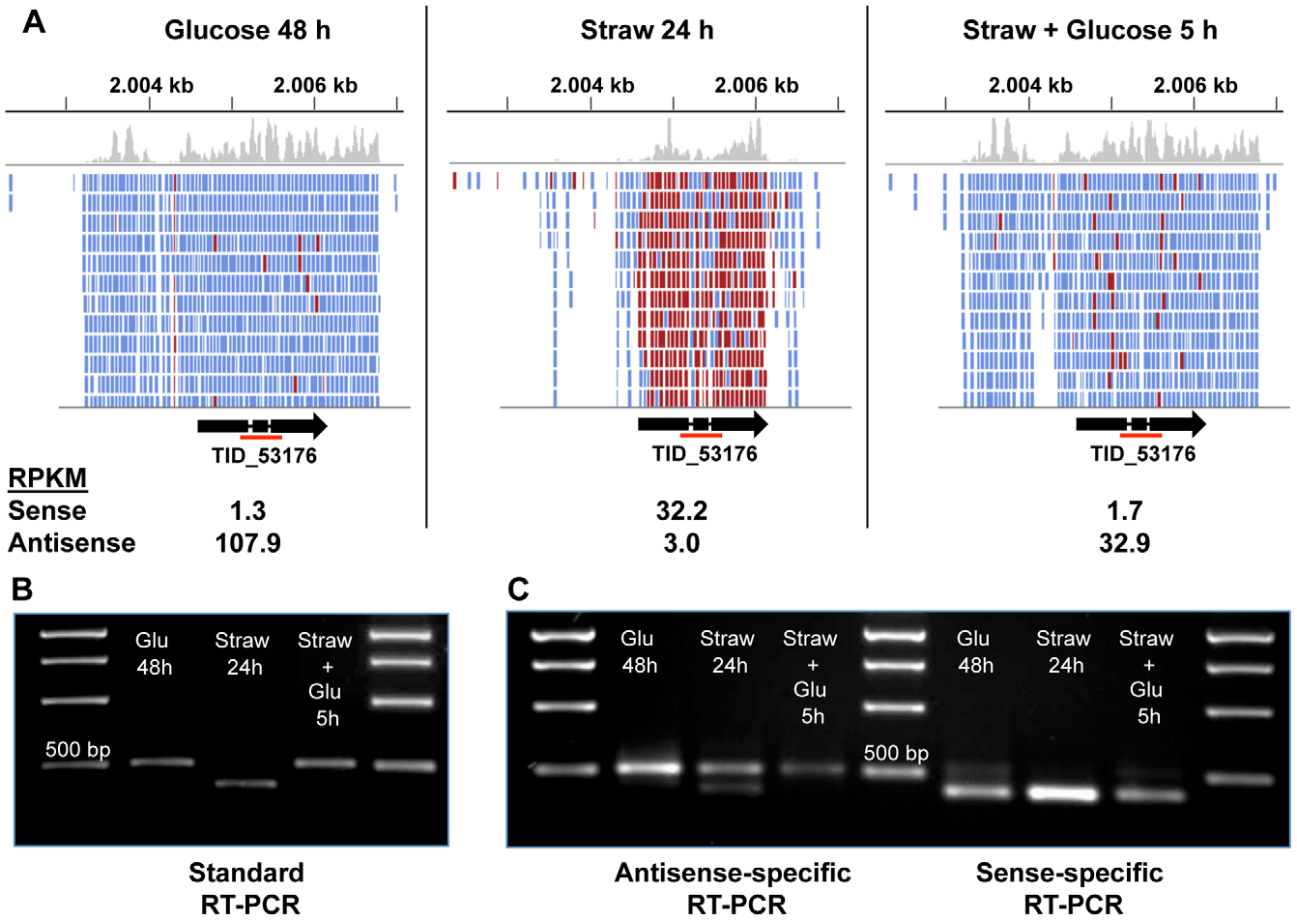
Sense and antisense transcription from TID_53176. (A) Alignment of RNA-seq reads to the TID_53176 genome region under each condition. Reads represented in blue are antisense, those in red are sense. The Figure was constructed using the Integrative Genomics Viewer [62]. (B) Oligo(dT) primed RT-PCR using TID_53176 specific primers. The expected band size from the spliced sense transcript is 411 bp and the size of the non-spliced antisense transcript is 524 bp. The red line under the gene model in panel A indicates the amplified region. (C) Strand-specific RT-PCR. One of the standard PCR primers, with an added sequence tag (Table S5), was used to synthesise cDNA from one strand only and then the PCR step was performed by using the tagged sequencing primer together with the opposing gene-specific primer. The larger band is only seen in the antisense-specific reaction, confirming it does represent an antisense transcript. The smaller band is the only band present in the sense-specific reaction.

In order to confirm that the NATs observed were biologically significant, and not simply an artefact of RNA-sequencing, a specific example, TID_53176, encoding a predicted membrane protein belonging to the GPR1/FUN34/YaaH family, which is required for acetate uptake in *A. nidulans* [53], was chosen for further analysis. The TID_53176 sense transcript is expressed in the straw media, whilst the AS transcript is expressed in the presence of glucose (Figure 4A).

The AS coverage level is high in both glucose conditions, and extends over the full length of the predicted gene including the two introns and extends both upstream and downstream, but does not overlap any neighbouring genes (Figure 4A). The sense coverage, seen in the straw condition, is shorter in length and there is almost zero coverage of the introns, indicating that the vast majority of sense transcripts are fully spliced. Figure 4B shows that RT-PCR, using primers upstream of the first intron and downstream of the second, can distinguish between the larger AS product and the shorter, fully spliced, sense transcript. Since oligo(dT) was used as the primer for cDNA synthesis, the AS transcript must be polyadenylated. To verify the strandedness of the two products, strand-specific RT-PCR was performed using a tagged primer approach and the results confirmed that all of the larger product is generated from antisense transcripts, whilst the smaller product is the only band seen in a sense-specific reaction (Figure 4C). A trace amount of a smaller antisense product can be seen in the antisense-specific assay under straw 24 h conditions. This may represent a true RNA intermediate, or it could be due to the high level of sense transcript self-priming and slight carryover of primer from the cDNA synthesis priming its amplification.

To identify AS transcripts that responded to the change in carbon source we calculated the ratio of antisense∶sense expression under Glucose 48 h and Straw 24 h conditions for the 521 genes with an AS RPKM of >1 (Table S4). Genes where sense transcription is induced on straw but AS predominates on glucose, include examples of transporters and permeases, CAZy enzymes and the putative lipase TID_173684.

This putative lipase TID_173684, one of the most highly induced genes upon exposure to straw, is also one of the genes showing the most marked antisense∶sense ratio switch (Table S4). Under glucose 48 h conditions there is significant expression of both sense and AS transcripts with a 60% greater level of AS (RPKMs of 1 and 1.6 respectively). After the switch to straw, at 24 h there is a large induction of the sense transcript (∼1700-fold) and AS transcription is cut to less than a third of the initial level (Figure S7A). Standard and strand-specific RT-PCR reactions under the same conditions give a similar pattern of bands to that seen above for TID_53176 (Figure 4). Interestingly, in the *ΔcreA* strain sense transcription is seen in both glucose and straw conditions, suggesting that the AS/S ratio switch is regulated either directly or indirectly by CreA. This was confirmed by strand-specific RT-PCR (Figure S7B). The timeline of induction experiment detailed earlier (Figure S1) shows that the AS/S switch in the expression of the putative lipase can be seen to occur between 3 and 6 h after the transfer to straw, which is concurrent with the expression of the carbon starvation induced subset of genes (of which the lipase gene is part). This suggests a possible relationship between carbon source responsive regulation by CreA and antisense transcription, providing an interesting area for further study.

## Methods

### Wheat straw analysis

#### Ball milling

The wheat straw (Cordiale variety) was milled using a Laboratory Mill (Laboratory Mill 3600, Perten, Sweden) and passed through a sieve with a mesh size of 700 µm for size reduction prior to ball milling. 5 g of pre-milled wheat straw was ball-milled in 80 mL stainless steel grinding bowls with 10-mm-diameter steel balls in a Planetary Mill (Pulverisette 5 classic line, Fritsch, Germany), at 400 rpm for a grinding time of 20 min, resulting in an average particle size of ∼75 µm.

#### Sugar analysis

The total sugar in processed ball-milled wheat straw was quantified in the hydrosylate after acid hydrolysis. 30 mg of dried wheat straw was weighed and subjected to a two stage acid hydrolysis initially with 12 M sulphuric acid for 1 hour at 37°C followed by 1 M sulphuric acid for 2 hours at 100°C. The monosaccharide analysis was performed on the supernatant. Sugar monomers in culture supernatants, collected at appropriate times points and separated from mycelia and insoluble straw by filtration through Miracloth (CalBioChem), and fully acid hydrolysed residues were determined by high-performance anion exchange chromatography with pulsed amperometric detection (HPAEC-PAD) (Dionex, UK) using a CarboPac PA20 column with 50 mM NaOH isocratic system at working flow rate of 0.5 ml/min at 30°C. Glucose, xylose, arabinose and galactose were used as standards with mannitol as an internal standard.

#### Lignin analysis

The acetyl bromide method [54], [55] was performed to quantify lignin in wheat straw samples. 100 mg of dried sample was digested with 4 ml of 25% v/v acetyl bromide in glacial acetic acid (HAc) in a Teflon capped tube at 50°C for 2 h with occasional mixing. After cooling, the digested sample volume was made up to 16 ml with HAc. After centrifugation at 3000 g for 15 min, 0.5 ml of the supernatant was mixed with 2.5 mL of HAc and 1.5 mL of 0.3M NaOH. 0.5 mL of 0.5M hydroxylamine hydrochloride solution was then added and the volume was made up to 10 mL with HAc. An analytical sample of low sulphate lignin (Sigma) was used as the standard for quantitative analysis by the acetyl bromide method. 10 mg of the lignin was dissolved in 5 ml of dioxane and 0.2, 0.3, 0.4, 0.5 and 0.6 mL was added in Teflon capped tubes to determine a standard curve. A reagent blank was also included. 0.5 mL of 25% acetyl bromide in glacial acetic acid (HAcBr) was added to each tube and incubated at 50°C for 30 min. The samples were cooled and 2.5 mL of HAc, 1.5 mL of 0.3 M NaOH, and 0.5 mL of 0.5 M hydroxylamine hydrochloride solution were added. The final volume was made up to 10 mL with HAc. Optical density was measured by scanning from 250 to 400 nm in a UV-Visible spectrophotometer (Varian, UK). The concentration of acetyl bromide soluble lignin for the samples was determined from the standard curve measured at 280 nm.

#### X-ray diffraction

The percentage cystallinity in the milled straw samples was estimated using X-ray diffraction, according to a variation of the method of Ruland and Vonk [56]. Cellulose is presumed to be the only crystallisable polymer in the cell-wall matrix so non-cellulose components such as lignin and hemicelluloses also contribute to the non-crystalline proportion. Powder measurements were carried out on a Siemens D5000 system, with copper Kα X-ray source, with scans performed from 5 to 50° 2è, A linear baseline was applied between the scan limits and a diffractogram of a ball-milled (fully amorphous) straw sample was then subtracted from the experimental profiles.

### Strains and growth conditions

The *A. niger* strains used were N402 [57] and AB4.1 *pyrG* [7] or as specified otherwise. Strains were maintained on potato dextrose agar (Oxoid). All AB4.1 cultures were supplemented by 10 mM uridine (Sigma). Cultures were incubated at 28°C until they had conidiated. Spores were harvested into 0.1% (v/v) Tween 20 (Sigma). Δ*xlnR* and Δ*creA* strains are *A. niger* AB4.1 *pyrG* containing a deletion of the respective open reading frame. Strains were constructed using the method developed by Scherer and Davis. [58] based on recombination between a plasmid containing the flanking region of the gene of interest and the chromosome. As a selection/counter-selection marker we used the gene coding for the orotidine-5-phosphate decarboxylase [59] (*pyrG*, from *Aspergillus oryzae*). After transformation of *A. niger*, cells were selected for uridine prototrophy, confirming integration of the plasmid into the chromosome. After purification of the transformants, release of the selective pressure for the integrated plasmid was achieved by propagating the clones twice on potato dextrose agar containing 10 mM uridine. Selection for cells that had excised the plasmid from the chromosome was done by plating them on media containing 4 mM of 5-fluoro-orotic acid (Melford) and 1.6 mM uridine. Deletion of *creA* or *xlnR* was confirmed by PCR using internal and external oligonucleotide primers and by sequencing around the respective loci.

Liquid batch cultures were inoculated with spores to a final concentration of 10^6^ spores/ml. *A. niger* was grown in 100 ml of minimal media [all l^−1^: NaNO_3_, 6 g; KCl, 0.52 g; MgSO_4_.7H_2_O, 0.52 g; KH_2_PO_4_, 1.52 g; Na_2_B_4_O_7_.10H_2_O, 0.008 mg; CuSO_4_.5H_2_O, 0.16 mg; FePO_4_.H_2_O, 0.16 mg; MnSO_4_.4H_2_O, 0.16 mg; NaMoO_4_.2H_2_O, 0.16 mg; ZnSO_4_, 1.6 mg] with the appropriate carbon source added to a final concentration of 1% (w/v) in 250 ml Erlenmeyer flasks at 28°C, shaken at 150 rpm. The standard time-course consisted of growth for 48 h in 1% (w/v) glucose media, after which mycelia were removed by filtration through Miracloth (Merck), washed thoroughly with media devoid of carbon source, and transferred to fresh media containing 1% (w/v) ball-milled wheat straw as sole carbon source. Incubation was continued for 24 h. Glucose was then added exogenously to a final concentration of 1% (w/v) and incubation continued for 5 hours. Figure 1 shows an image of a mycelial clump that was magnified using a Nikon SMZ1000 stereomicroscope and the picture taken using a Nikon 4500 camera.

### RNA extraction

Mycelia from each condition were frozen and ground under liquid nitrogen using a mortar and pestle. Total RNA was extracted from the ground material using the TRIzol reagent protocol (Invitrogen). An additional clean-up was performed using the RNEasy Mini Kit (Qiagen), following the manufacturer's RNA Clean-up protocol, including the additional on-column DNAse digest.

### RT-PCR

SuperScript III Reverse Transcriptase (Invitrogen) was used to synthesise cDNA from total RNA according to manufacturer's instructions, using oligo(dT) as primer or tagged gene specific primers for strand-specific experiments. 0.5 µg of total RNA was used for each reverse transcription. PCR reactions were performed using Phusion (New England Biolabs) using 1 µl of cDNA in a 25 µl reaction. PCR conditions were 30 cycles of denaturation at 98°C for 30 s, annealing at 60°C for 30 s and extension at 72°C for 30 s. For PCR amplification of cDNA synthesised strand-specifically, a primer identical to the added tag sequence was paired with the opposing standard primer. In this way only cDNA synthesised from the tagged primer, and not non-specifically self-primed transcripts, was amplified.

qPCR amplifications were carried out using the Applied Biosystems 7500 Fast Real-Time PCR system. The PCR reaction mixture (10 µl) contained 1 µl of cDNA, specific primer sets (175 nM final concentration), and FAST SYBR-Green Master Mix (Applied Biosystems). PCRs were carried out for 40 cycles; denaturation at 95°C for 15 seconds, annealing at 67°C for 30 seconds, and extension at 60°C for 60 seconds. All measurements were independently conducted 3 times on 2 separate biological isolates. The specificity of primer sets used for qRT-PCR amplification was evaluated by melting curve analysis. The Standard Curve Method was used for quantification against a known concentration of genomic DNA (Li et al 2009). All primers used, and sequences are listed in Table S5.

### RNA-seq

10 µg of Total RNA was depleted of ribosomal RNA using the Ribominus Eukaryotic kit (Invitrogen). SOLiD whole transcriptome libraries were made as outlined in the SOLiD Whole transcriptome kit protocol (Applied Biosystems). The Quant-it HS dsDNA assay kit (Invitrogen) was used to measure the concentration of libraries in order to pool equimolar amounts. Pooled libraries were gel-purified using 2% size-select E-gels to 200–300 bp (Invitrogen). Emulsion PCR (0.5 pM final concentration of pooled libraries) and bead-based enrichment was carried out according to the SOLiD 4 Templated bead preparation guide containing library. Sequencing was performed on a SOLiD 4 ABi sequencer according to the manufacturer's instructions to generate 50 bp reads in colour space.

### Data analysis

SOLiD 4 RNA-seq reads from each experimental sample were mapped to the two published genome sequences of *A. niger* (JGI *A. niger* version 3.0 unmasked assembly scaffolds and gene annotation and CADRE *A. niger* assembly) using the BioScope 1.3.1 Whole Transcriptome Pipeline (LifeTechnologies). This included the initial filtering of reads against a collection of published *A. niger* rRNA sequences prior to mapping (Genbank [60] sequence IDs: 197115286, 300675560, 222105557, 312434471, 34304202, 241017633, 157267321, 213866863). BioScope provided the primary read alignment position of each read mapped against the complete genome sequence and exon spanning junctions using available gene coordinate information. Read alignment results were recorded in BAM format for further downstream analysis. Read counts per gene were calculated for each sample with Htseq-count (http://www-huber.embl.de/users/anders/HTSeq) using the BAM file and genome annotation as the input. Strand-specific RNA-seq reads, as generated by SOLiD, can be specified for when executing Htseq-count in determining accurate read counts per gene. These counts were then used to calculate normalized expression values (RPKM) for each gene [61]. Additionally, antisense transcription could be detected by comparing gene counts generated by Htseq-count when opting to ignore or include strand-specificity in the calculations.. Data were further visualised using Integrative Genomic Viewer (IGV) [62]. Raw and processed data files have been submitted to the Gene Expression Omnibus, under accession number GSE33852. Reads were mapped against an EST assembly of Genbank EST records [60] for the wheat *Triticum aestivum* (taxon id 4565; 1,073,845 EST's March 2012) a publicly available wheat genome [63]. The number of reads that mapped was no higher in the wheat-grown *A. niger* cultures than it was in the glucose-grown cultures. This suggests wheat transcript levels are insignificant, and any reads mapping to the wheat genome are simply due to a basic level of homology between genomes.

### Identification of secreted proteins

Proteins in culture supernatant from relevant times were separated on 4–20% Tris-glycine Novex SDS-PAGE gels (Invitrogen). 15 µl Tris-glycine-SDS loading buffer was added to 12 µl supernatant plus 3 µl 1 M DTT. The sample mixture was heated to 100°C for 5 minutes before being loaded onto the gel with running conditions of 100 V for 3 hours. The gel was subsequently silver stained [64]. Bands resolved by size were excised from the gel, diced into cubes (∼1 mm^3^) and placed into individual wells of a microtitre plate, processed (destained, reduced, alkylated) and digested with trypsin using standard procedures on a MassPREP station (Waters). Resulting peptides were delivered via nanoflow reversed phase-HPLC to a Q-ToF2 (Waters) for tandem MS analysis. An automated experiment (DDA = data dependent acquisition) was run where selected peptides automatically enter MSMS for fragmentation. The data was searched against the Swissprot and NCBInr databases using the MS/MSIONS search on the MASCOT web site (http://www.matrixscience.com) using the variable modifications of carbamidomethylation of cysteine and oxidation of methionine.

## Supporting Information

Figure S1 **RT-PCR measurement of gene expression over time after the switch to growth on wheat straw.** Results shown are representative of at least two biologically independent experiments. Time of induction varies for different genes, with two notably distinct clusters of induction at the 6 h and 9 h time points.
(PDF)

Figure S2 **qRT-PCR analysis of glycoside hydrolase gene expression after 6 and 9 hours of exposure to straw.** Expression was measured in the N402 strain. Results shown are representative of at least two biologically independent experiments. * indicates significant induction (a p-value of >0.01 in an equal variance, one-tailed T-test) compared to the expression level measured at the Glucose 48 h time point.
(PDF)

Figure S3 **qRT-PCR analysis of glycoside hydrolase gene expression after 24 hours exposure to straw in** *ΔxlnR* **and parent strain.** Results shown are triplicate measurements taken from each of at least two biologically independent experiments.
(PDF)

Figure S4 qRT-PCR analysis of glycoside hydrolase gene expression in the *ΔcreA* **and parent strain after 48 hours growth on glucose.** Results shown are triplicate measurements taken from each of at least two biologically independent experiments. * indicates significant change in expression level (a p-value of >0.01 in an equal variance, one-tailed T-test) in the mutant strain, when compared to the parent strain.
(PDF)

Figure S5 **qRT-PCR analysis of glycoside hydrolase gene expression in the N402 wild-type after the switch to media completely devoid of carbon source.** Expression was measured in the wild-type strain. Results shown are representative of at least two biologically independent experiments. Fold-induction values for *cbhA* and *GH61* inductions were <3 and are therefore not visible on the scale of this Figure. * indicates significant induction (a p-value of >0.01 in an equal variance, one-tailed T-test) compared to the expression level measured at the Glucose 48 h time point.
(PDF)

Figure S6 **Distribution of genes as a function of their antisense RPKM.** Antisense RPKM was calculated for each gene in Glucose 48 h (blue bar) and Straw 24 h (red bar). Approximately 5 percent of genes have a value of 1 or more RPKM. The number of genes is represented on a log scale.
(PDF)

Figure S7 **Characterisation of** *tfl1* **transcripts from sense and antisense directions and their regulation by CreA.** **A. Antisense transcription through** *tfl1* **in glucose growth conditions.** RNA-sequencing reads aligned to the *tfl1* locus in Glucose 48 h and Straw 24 h conditions. The figure is adapted from Integrated Genomics Viewer. Blue reads are sense, and red reads antisense, to *tfl1*. RPKM values for sense and antisense for each condition are given below the gene model. The red line below the gene model indicates the region amplified in RT-PCR (B). **B. Confirmation of antisense transcript and its regulation by CreA.** The RT-PCR primers amplify a region (indicated by the red line) spanning across the first two introns of the gene (A). A non-spliced transcript will generate a product of 507 bp; A fully spliced transcript, a product of 365 bp, and a transcript with a single intron removed will give a product of either 421 or 451 bp. Standard RT-PCR (which does not differentiate between sense and antisense transcripts) on the parent strain shows, when grown on straw the product size is as expected from a spliced transcript template. In contrast, when grown on glucose, a larger product is observed. Strand-specific RT-PCR confirms that the predominant band seen at glucose 48 h in the conventional RT-PCR is from the antisense strand, whilst in straw the predominant band is sense to *tfl1*. In a *ΔcreA* strain, standard and strand-specific RT-PCR shows that the products formed are from RNAs that are mainly sense to *tfl1*. The existence of low levels of spliced and partially spliced intermediates of antisense transcripts can also be observed. Results shown are representative of at least two biologically independent experiments.
(PDF)

Table S1 **Genes annotated in the CBS 513.8 genome but not the ATCC 1015 genome.**
(XLS)

Table S2 **Read mapping scores and RPKM values for each biological replicate, the combined mapping scores and statistical significance scores for all genes under Glucose 48 h, Straw 24 h and Straw+Glucose 5 h conditions.**
(XLSX)

Table S3 **Inter-sample correlation comparison.** R-squared values for comparison of each of the mapping results of each individual biological sample.
(XLSX)

Table S4 **Antisense and sense RPKM values and ratios under Glucose 48 h, Straw 24 h and Straw+Glucose 5 h conditions, for all genes with an antisense RPKM >1 in any condition.**
(XLSX)

Table S5 **Primers used in this study.**
(XLSX)
