# Supplementary figures and images for: Uncovering the Genome-Wide Transcriptional Responses of the Filamentous Fungus Aspergillus niger to Lignocellulose Using RNA Sequencing
Source: PLoS Genet. 2012 Aug 9;8(8):e1002875. doi: 10.1371/journal.pgen.1002875 (PMC3415456; doi:10.1371/journal.pgen.1002875)

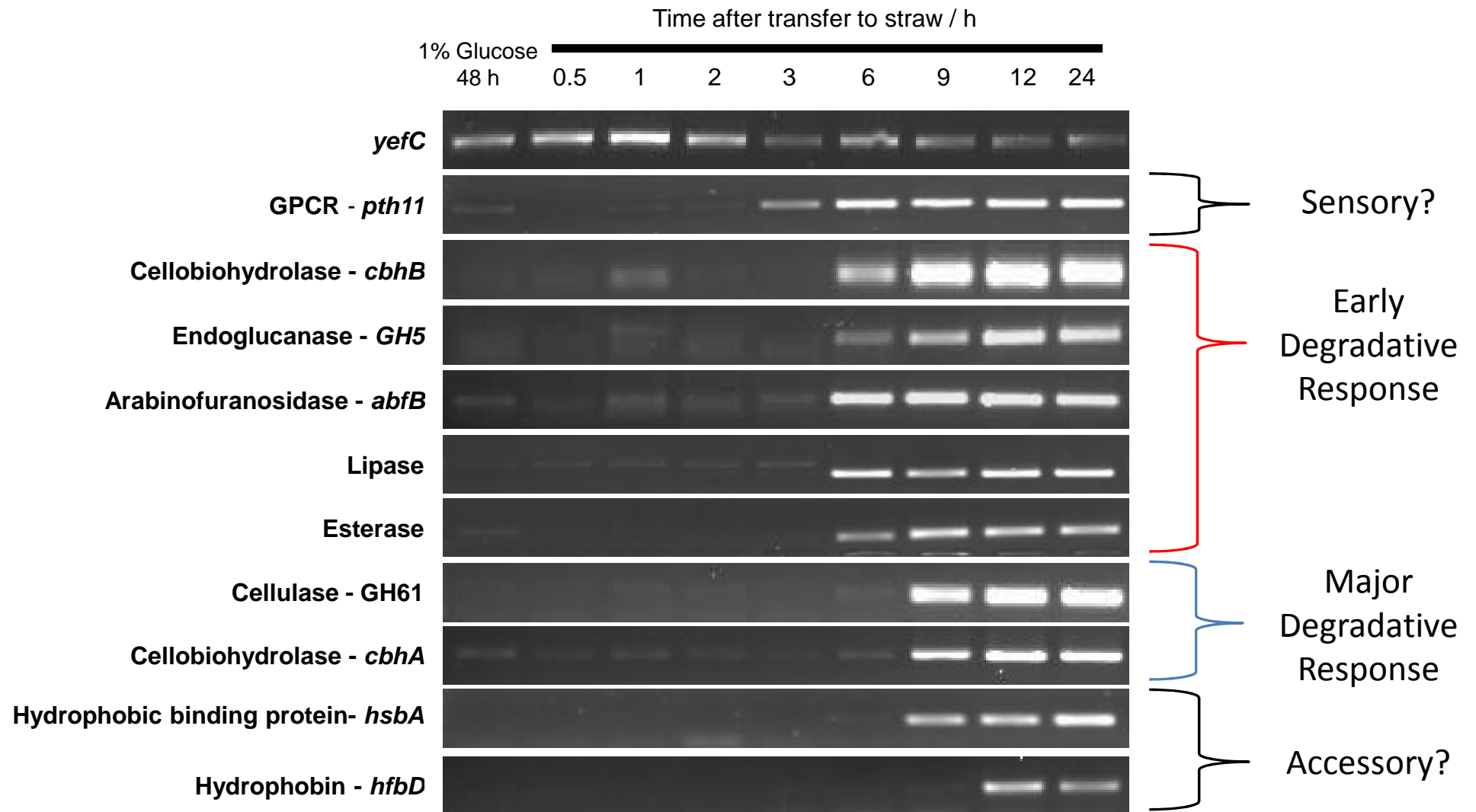

Supplement: Figure S1 — RT-PCR measurement of gene expression over time after the switch to growth on wheat straw. Results shown are representative of at least two biologically independent experiments. Time of induction varies for different genes, with two notably distinct clusters of induction at the 6 h and 9 h time points. (PDF) [file pgen.1002875.s001.pdf]

Fold change relative to glucose 48 h sample

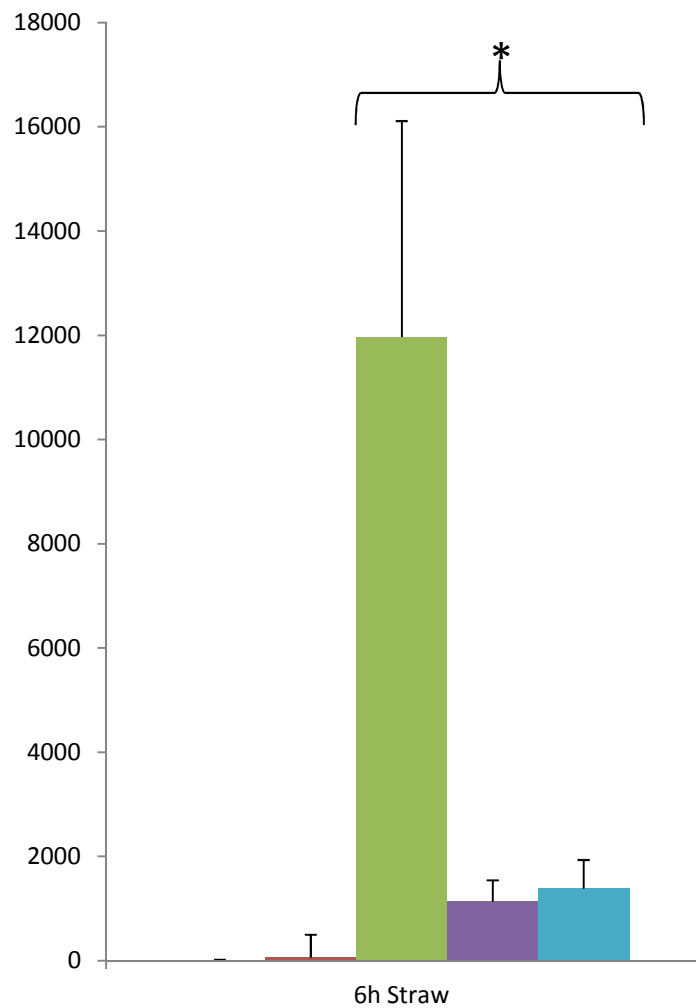

Fold change relative to glucose 48 h sample

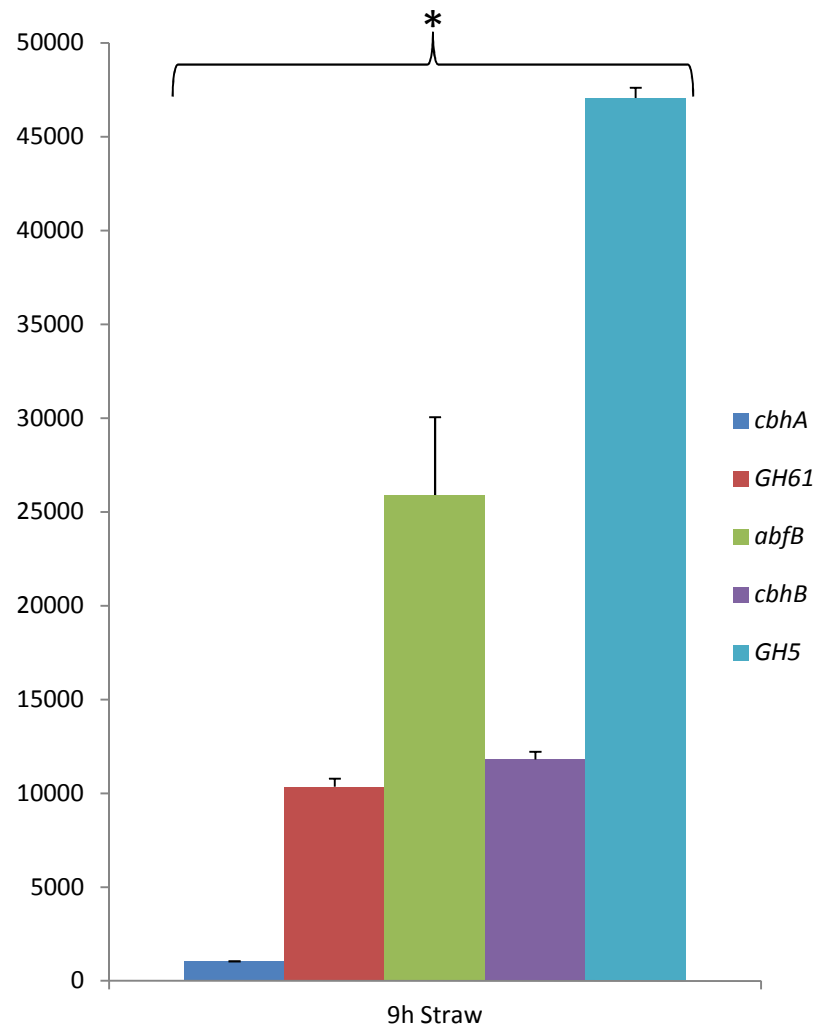

Supplement: Figure S2 — qRT-PCR analysis of glycoside hydrolase gene expression after 6 and 9 hours of exposure to straw. Expression was measured in the N402 strain. Results shown are representative of at least two biologically independent experiments. * indicates significant induction (a p-value of >0.01 in an equal variance, one-tailed T-test) compared to the expression level measured at the Glucose 48 h time point. (PDF) [file pgen.1002875.s002.pdf]

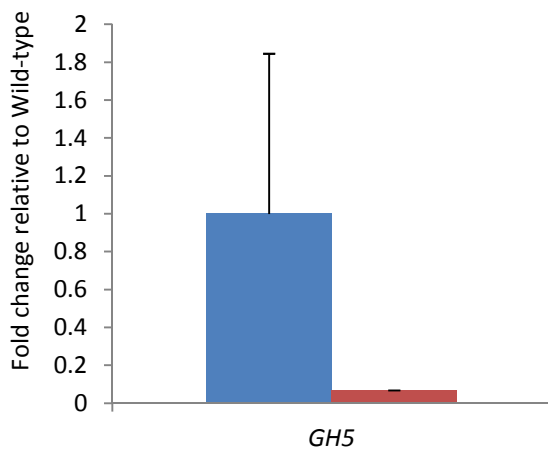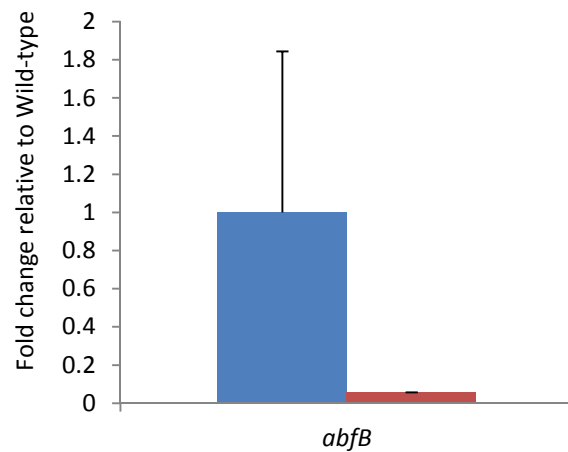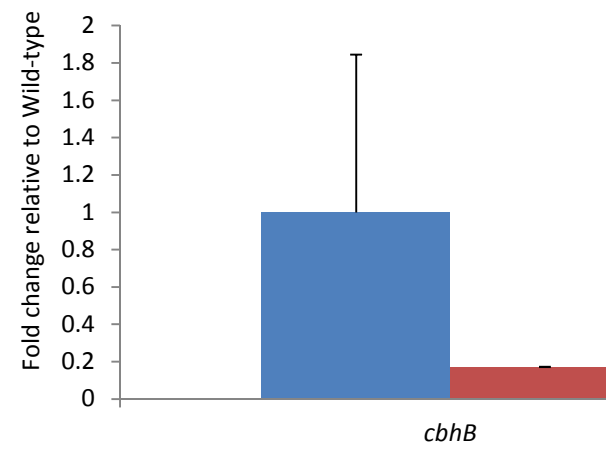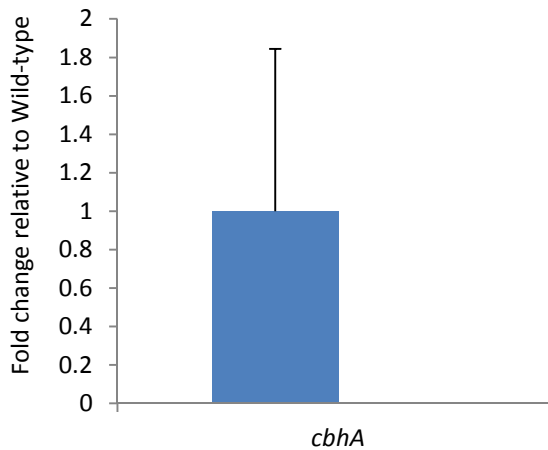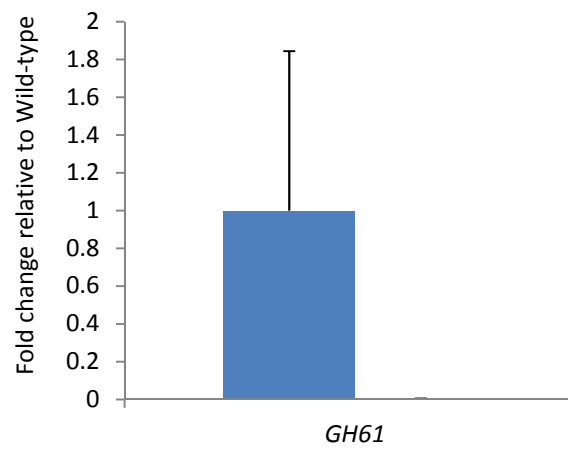

■ Straw 24 h Wild-type  
■ Straw 24 h  $\Delta xlnR$

Supplement: Figure S3 — qRT-PCR analysis of glycoside hydrolase gene expression after 24 hours exposure to straw in ΔxlnR and parent strain. Results shown are triplicate measurements taken from each of at least two biologically independent experiments. (PDF) [file pgen.1002875.s003.pdf]

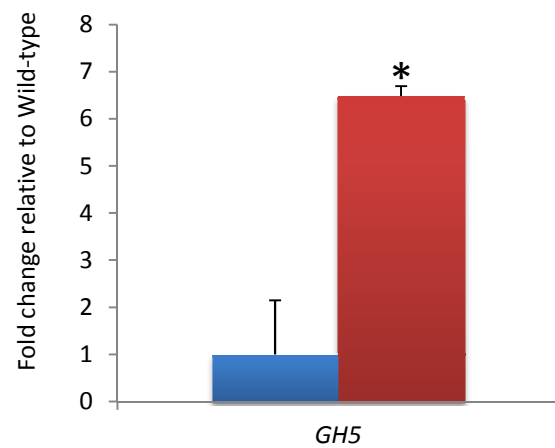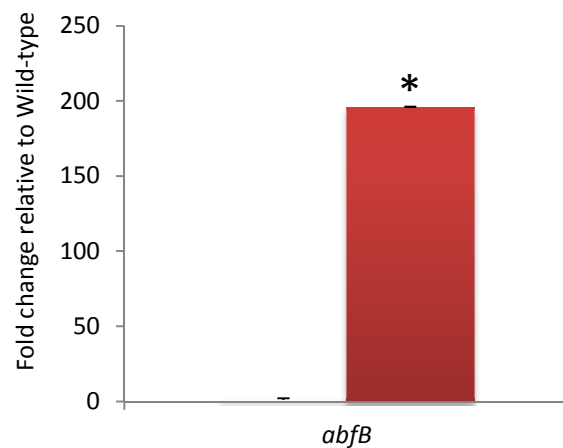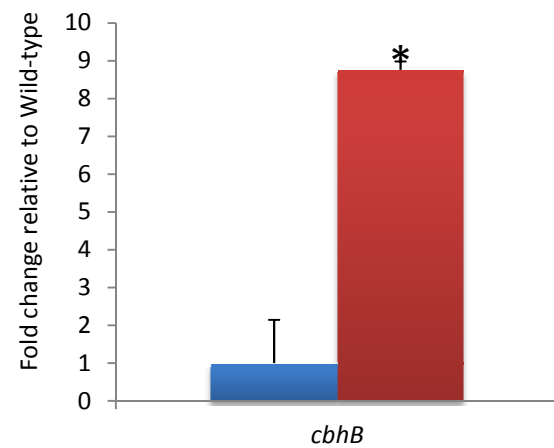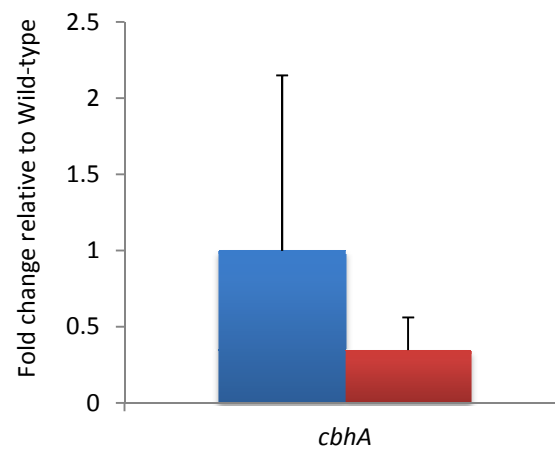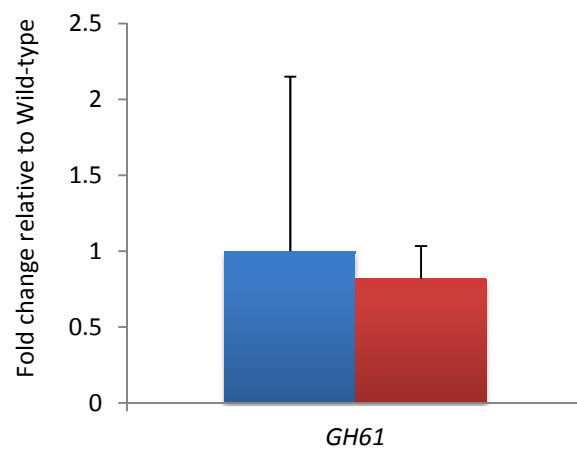

■ Glucose 48 h Wild-type  
■ Glucose 48 h  $\Delta creA$

Supplement: Figure S4 — qRT-PCR analysis of glycoside hydrolase gene expression in the ΔcreA and parent strain after 48 hours growth on glucose. Results shown are triplicate measurements taken from each of at least two biologically independent experiments. * indicates significant change in expression level (a p-value of >0.01 in an equal variance, one-tailed T-test) in the mutant strain, when compared to the parent strain. (PDF) [file pgen.1002875.s004.pdf]

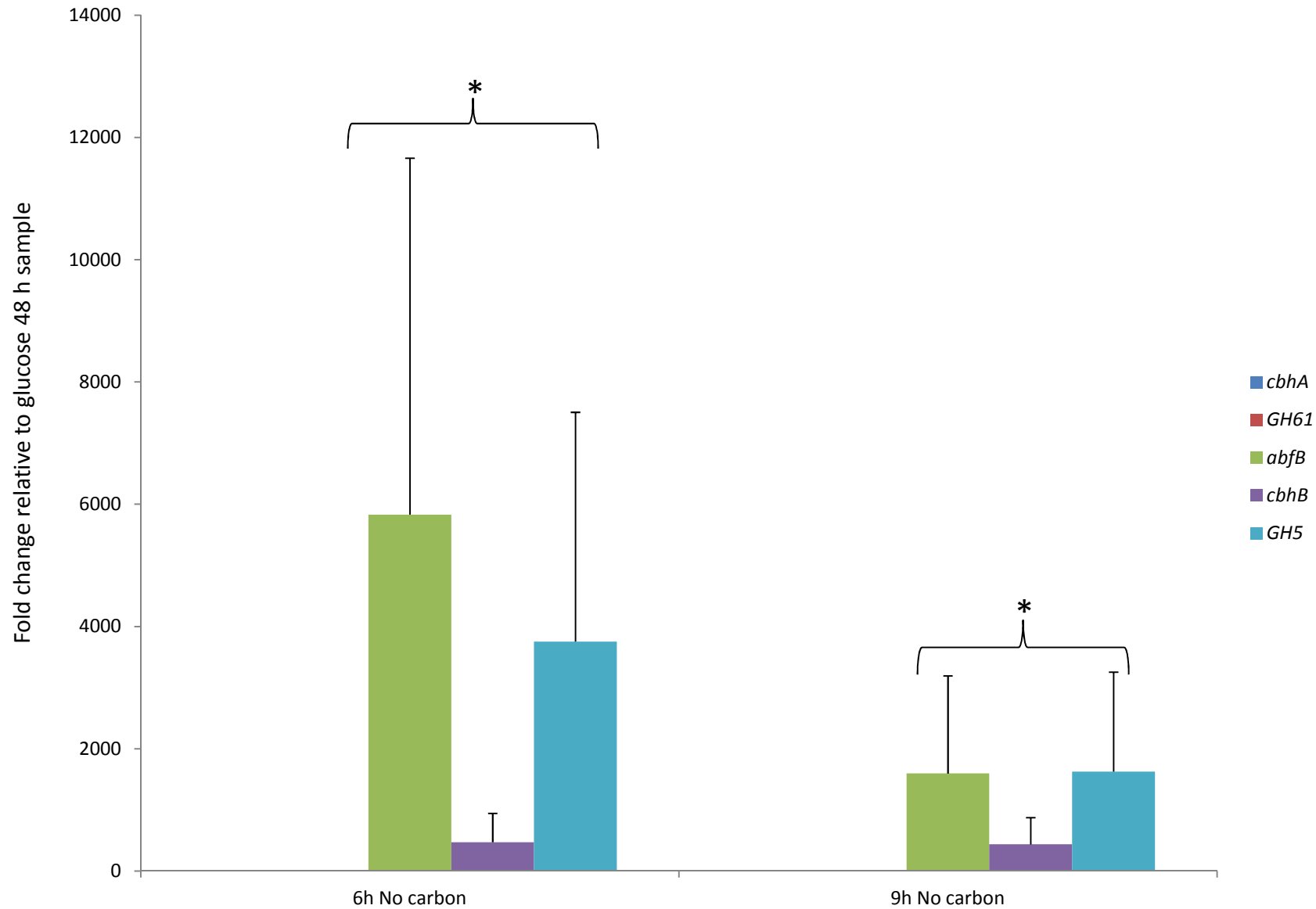

Supplement: Figure S5 — qRT-PCR analysis of glycoside hydrolase gene expression in the N402 wild-type after the switch to media completely devoid of carbon source. Expression was measured in the wild-type strain. Results shown are representative of at least two biologically independent experiments. Fold-induction values for cbhA and GH61 inductions were <3 and are therefore not visible on the scale of this Figure. * indicates significant induction (a p-value of >0.01 in an equal variance, one-tailed T-test) compared to the expression level measured at the Glucose 48 h time point. (PDF) [file pgen.1002875.s005.pdf]

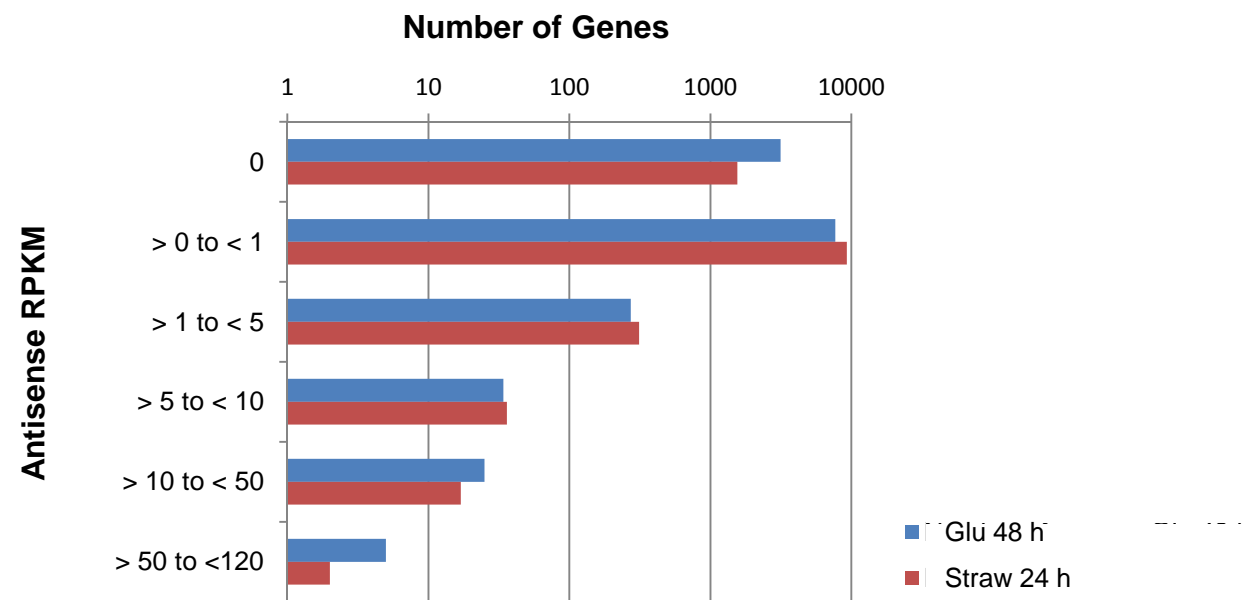

Supplement: Figure S6 — Distribution of genes as a function of their antisense RPKM. Antisense RPKM was calculated for each gene in Glucose 48 h (blue bar) and Straw 24 h (red bar). Approximately 5 percent of genes have a value of 1 or more RPKM. The number of genes is represented on a log scale. (PDF) [file pgen.1002875.s006.pdf]
