# Supplementary material for: Uncovering the Genome-Wide Transcriptional Responses of the Filamentous Fungus Aspergillus niger to Lignocellulose Using RNA Sequencing
Source: PLoS Genet. 2012 Aug 9;8(8):e1002875. doi: 10.1371/journal.pgen.1002875 (PMC3415456; doi:10.1371/journal.pgen.1002875)
Supplement: Figure S7 — Characterisation of tfl1 transcripts from sense and antisense directions and their regulation by CreA. A. Antisense transcription through tfl1 in glucose growth conditions. RNA-sequencing reads aligned to the tfl1 locus in Glucose 48 h and Straw 24 h conditions. The figure is adapted from Integrated Genomics Viewer. Blue reads are sense, and red reads antisense, to tfl1. RPKM values for sense and antisense for each condition are given below the gene model. The red line below the gene model indicates the region amplified in RT-PCR (B). B. Confirmation of antisense transcript and its regulation by CreA. The RT-PCR primers amplify a region (indicated by the red line) spanning across the first two introns of the gene (A). A non-spliced transcript will generate a product of 507 bp; A fully spliced transcript, a product of 365 bp, and a transcript with a single intron removed will give a product of either 421 or 451 bp. Standard RT-PCR (which does not differentiate between sense and antisense transcripts) on the parent strain shows, when grown on straw the product size is as expected from a spliced transcript template. In contrast, when grown on glucose, a larger product is observed. Strand-specific RT-PCR confirms that the predominant band seen at glucose 48 h in the conventional RT-PCR is from the antisense strand, whilst in straw the predominant band is sense to tfl1. In a ΔcreA strain, standard and strand-specific RT-PCR shows that the products formed are from RNAs that are mainly sense to tfl1. The existence of low levels of spliced and partially spliced intermediates of antisense transcripts can also be observed. Results shown are representative of at least two biologically independent experiments. (PDF) [file pgen.1002875.s007.pdf]

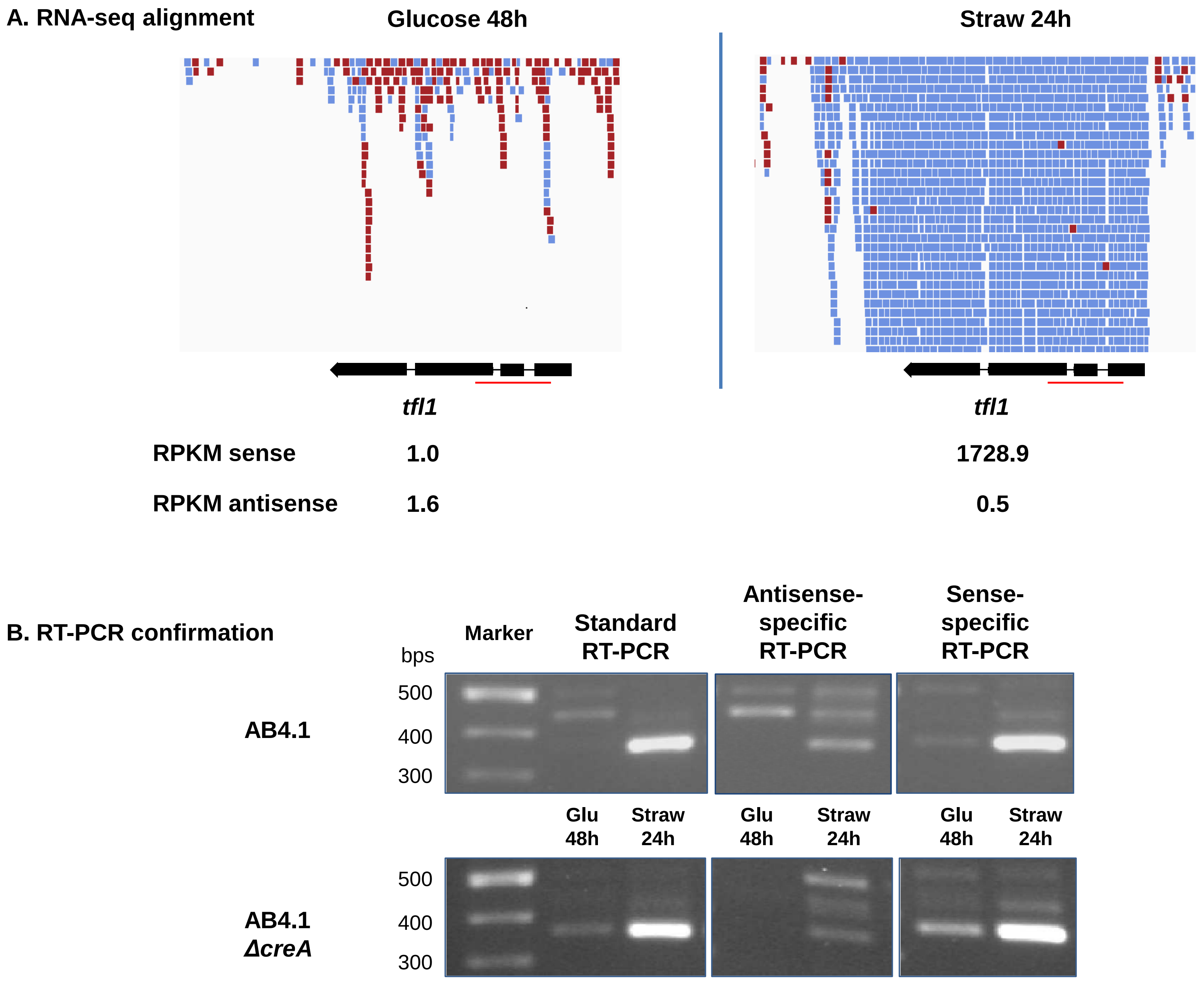
A. RNA-seq alignment
Glucose 48h
Straw 24h
tfl1
tfl1
RPKM sense
1.0
1728.9
RPKM antisense
1.6
0.5
B. RT-PCR confirmation
Marker
Standard RT-PCR
Antisense-specific RT-PCR
Sense-specific RT-PCR
bps
500
400
300
AB4.1
Glu 48h
Straw 24h
Glu 48h
Straw 24h
Glu 48h
Straw 24h
500
400
300
AB4.1 ΔcreA
